# Supplementary material for: Unveiling the Bioactive Potential of the Invasive Jellyfish Phyllorhiza punctata Through Integrative Transcriptomic and Proteomic Analyses
Source: Biomolecules. 2025 Aug 4;15(8):1121. doi: 10.3390/biom15081121 (PMC12383608; doi:10.3390/biom15081121)
Supplement: Supplementary file 1 [file biomolecules-15-01121-s001.zip › Suplementary Material/FileS3/PEPFOLD4/XP_065062955_14706/clusters.html]

 PEP-FOLD clusters


Download archive of all models  here   

|  |  |  |
| --- | --- | --- |
| # | file | sOPEP |
| # cluster 1 - Size: 6 |  |  |
|  | XP\_065062955\_14706-model1 | -13.0423 |
|  | XP\_065062955\_14706-model1.1 | -11.9864 |
|  | XP\_065062955\_14706-model1.2 | -11.2306 |
|  | XP\_065062955\_14706-model1.3 | -10.9735 |
|  | XP\_065062955\_14706-model1.4 | -10.108 |
|  | XP\_065062955\_14706-model1.5 | -9.9996 |
| # cluster 2 - Size: 13 |  |  |
|  | XP\_065062955\_14706-model2 | -12.8258 |
|  | XP\_065062955\_14706-model2.1 | -12.0674 |
|  | XP\_065062955\_14706-model2.2 | -12.0563 |
|  | XP\_065062955\_14706-model2.3 | -12.041 |
|  | XP\_065062955\_14706-model2.4 | -11.9349 |
|  | XP\_065062955\_14706-model2.5 | -11.9134 |
|  | XP\_065062955\_14706-model2.6 | -11.8139 |
|  | XP\_065062955\_14706-model2.7 | -11.7376 |
|  | XP\_065062955\_14706-model2.8 | -11.5523 |
|  | XP\_065062955\_14706-model2.9 | -11.4751 |
|  | XP\_065062955\_14706-model2.10 | -11.2248 |
|  | XP\_065062955\_14706-model2.11 | -11.2039 |
|  | XP\_065062955\_14706-model2.12 | -10.3351 |
| # cluster 3 - Size: 1 |  |  |
|  | XP\_065062955\_14706-model3 | -11.5465 |
| # cluster 4 - Size: 9 |  |  |
|  | XP\_065062955\_14706-model4 | -11.312 |
|  | XP\_065062955\_14706-model4.1 | -11.2939 |
|  | XP\_065062955\_14706-model4.2 | -11.0752 |
|  | XP\_065062955\_14706-model4.3 | -10.6879 |
|  | XP\_065062955\_14706-model4.4 | -10.6859 |
|  | XP\_065062955\_14706-model4.5 | -10.4515 |
|  | XP\_065062955\_14706-model4.6 | -10.381 |
|  | XP\_065062955\_14706-model4.7 | -10.1906 |
|  | XP\_065062955\_14706-model4.8 | -9.84633 |
| # cluster 5 - Size: 4 |  |  |
|  | XP\_065062955\_14706-model5 | -10.8453 |
|  | XP\_065062955\_14706-model5.1 | -10.4079 |
|  | XP\_065062955\_14706-model5.2 | -10.2344 |
|  | XP\_065062955\_14706-model5.3 | -10.1728 |
| # cluster 6 - Size: 6 |  |  |
|  | XP\_065062955\_14706-model6 | -10.8044 |
|  | XP\_065062955\_14706-model6.1 | -10.5924 |
|  | XP\_065062955\_14706-model6.2 | -10.2018 |
|  | XP\_065062955\_14706-model6.3 | -10.1787 |
|  | XP\_065062955\_14706-model6.4 | -10.1602 |
|  | XP\_065062955\_14706-model6.5 | -10.1034 |
| # cluster 7 - Size: 3 |  |  |
|  | XP\_065062955\_14706-model7 | -10.7164 |
|  | XP\_065062955\_14706-model7.1 | -10.203 |
|  | XP\_065062955\_14706-model7.2 | -9.95064 |
| # cluster 8 - Size: 1 |  |  |
|  | XP\_065062955\_14706-model8 | -10.7052 |
| # cluster 9 - Size: 7 |  |  |
|  | XP\_065062955\_14706-model9 | -10.6975 |
|  | XP\_065062955\_14706-model9.1 | -10.4753 |
|  | XP\_065062955\_14706-model9.2 | -10.3938 |
|  | XP\_065062955\_14706-model9.3 | -10.3516 |
|  | XP\_065062955\_14706-model9.4 | -10.2415 |
|  | XP\_065062955\_14706-model9.5 | -10.1955 |
|  | XP\_065062955\_14706-model9.6 | -10.1906 |
| # cluster 10 - Size: 4 |  |  |
|  | XP\_065062955\_14706-model10 | -10.6788 |
|  | XP\_065062955\_14706-model10.1 | -10.561 |
|  | XP\_065062955\_14706-model10.2 | -10.3581 |
|  | XP\_065062955\_14706-model10.3 | -10.3164 |
| # cluster 11 - Size: 5 |  |  |
|  | XP\_065062955\_14706-model11 | -10.4734 |
|  | XP\_065062955\_14706-model11.1 | -10.4727 |
|  | XP\_065062955\_14706-model11.2 | -10.2648 |
|  | XP\_065062955\_14706-model11.3 | -10.2537 |
|  | XP\_065062955\_14706-model11.4 | -9.97215 |
| # cluster 12 - Size: 5 |  |  |
|  | XP\_065062955\_14706-model12 | -10.4644 |
|  | XP\_065062955\_14706-model12.1 | -10.4366 |
|  | XP\_065062955\_14706-model12.2 | -10.4288 |
|  | XP\_065062955\_14706-model12.3 | -9.94304 |
|  | XP\_065062955\_14706-model12.4 | -9.8774 |
| # cluster 13 - Size: 2 |  |  |
|  | XP\_065062955\_14706-model13 | -10.4454 |
|  | XP\_065062955\_14706-model13.1 | -10.1669 |
| # cluster 14 - Size: 7 |  |  |
|  | XP\_065062955\_14706-model14 | -10.4238 |
|  | XP\_065062955\_14706-model14.1 | -10.2304 |
|  | XP\_065062955\_14706-model14.2 | -10.1499 |
|  | XP\_065062955\_14706-model14.3 | -10.1111 |
|  | XP\_065062955\_14706-model14.4 | -10.0342 |
|  | XP\_065062955\_14706-model14.5 | -10.0161 |
|  | XP\_065062955\_14706-model14.6 | -9.9382 |
| # cluster 15 - Size: 2 |  |  |
|  | XP\_065062955\_14706-model15 | -10.4146 |
|  | XP\_065062955\_14706-model15.1 | -10.0478 |
| # cluster 16 - Size: 3 |  |  |
|  | XP\_065062955\_14706-model16 | -10.3904 |
|  | XP\_065062955\_14706-model16.1 | -10.2385 |
|  | XP\_065062955\_14706-model16.2 | -10.1914 |
| # cluster 17 - Size: 7 |  |  |
|  | XP\_065062955\_14706-model17 | -10.3899 |
|  | XP\_065062955\_14706-model17.1 | -10.3367 |
|  | XP\_065062955\_14706-model17.2 | -10.2562 |
|  | XP\_065062955\_14706-model17.3 | -10.2284 |
|  | XP\_065062955\_14706-model17.4 | -10.1551 |
|  | XP\_065062955\_14706-model17.5 | -10.1535 |
|  | XP\_065062955\_14706-model17.6 | -10.1307 |
| # cluster 18 - Size: 2 |  |  |
|  | XP\_065062955\_14706-model18 | -10.3785 |
|  | XP\_065062955\_14706-model18.1 | -9.81982 |
| # cluster 19 - Size: 2 |  |  |
|  | XP\_065062955\_14706-model19 | -10.2313 |
|  | XP\_065062955\_14706-model19.1 | -9.82298 |
| # cluster 20 - Size: 1 |  |  |
|  | XP\_065062955\_14706-model20 | -10.2292 |
| # cluster 21 - Size: 2 |  |  |
|  | XP\_065062955\_14706-model21 | -10.1861 |
|  | XP\_065062955\_14706-model21.1 | -10.1334 |
| # cluster 22 - Size: 4 |  |  |
|  | XP\_065062955\_14706-model22 | -10.1711 |
|  | XP\_065062955\_14706-model22.1 | -10.0849 |
|  | XP\_065062955\_14706-model22.2 | -10.023 |
|  | XP\_065062955\_14706-model22.3 | -9.85129 |
| # cluster 23 - Size: 2 |  |  |
|  | XP\_065062955\_14706-model23 | -10.1632 |
|  | XP\_065062955\_14706-model23.1 | -9.79698 |
| # cluster 24 - Size: 1 |  |  |
|  | XP\_065062955\_14706-model24 | -10.0287 |
| # cluster 25 - Size: 1 |  |  |
|  | XP\_065062955\_14706-model25 | -9.99199 |
|  |  |  |
